# Neuroprotective effect of remote ischemic preconditioning in patients undergoing cardiac surgery: A randomized controlled trial

**DOI:** 10.3389/fcvm.2022.952033

**Published:** 2022-09-06

**Authors:** Shouqiang Zhu, Ziyu Zheng, Wenying Lv, Pengrong Ouyang, Jiange Han, Jiaqiang Zhang, Hailong Dong, Chong Lei

**Affiliations:** ^1^Department of Anesthesiology and Perioperative Medicine, Xijing Hospital, Air Force Medical University, Xi’an, China; ^2^Department of Anesthesiology, Tianjin Chest Hospital, Tianjin, China; ^3^Department of Anesthesiology and Perioperative Medicine, Henan Provincial People’s Hospital, People’s Hospital of Zhengzhou University, Zhengzhou, China

**Keywords:** neuroprotective effect, remote ischemic preconditioning, cardiac surgery, surrogate, postoperative cognitive dysfunction

## Abstract

**Background:**

The neuroprotective effect of remote ischemic preconditioning (RIPC) in patients undergoing elective cardiopulmonary bypass (CPB)-assisted coronary artery bypass graft (CABG) or valvular cardiac surgery remains unclear.

**Methods:**

A randomized, double-blind, placebo-controlled superior clinical trial was conducted in patients undergoing elective on-pump coronary artery bypass surgery or valve surgery. Before anesthesia induction, patients were randomly assigned to RIPC (three 5-min cycles of inflation and deflation of blood pressure cuff on the upper limb) or the control group. The primary endpoint was the changes in S-100 calcium-binding protein β (S100-β) levels at 6 h postoperatively. Secondary endpoints included changes in Neuron-specific enolase (NSE), Mini-mental State Examination (MMSE), and Montreal Cognitive Assessment (MoCA) levels.

**Results:**

A total of 120 patients [mean age, 48.7 years; 36 women (34.3%)] were randomized at three cardiac surgery centers in China. One hundred and five patients were included in the modified intent-to-treat analysis (52 in the RIPC group and 53 in the control group). The primary result demonstrated that at 6 h after surgery, S100-β levels were lower in the RIPC group than in the control group (50.75; 95% confidence interval, 67.08 to 64.40 pg/ml vs. 70.48; 95% CI, 56.84 to 84.10 pg/ml, *P* = 0.036). Compared to the control group, the concentrations of S100-β at 24 h and 72 h and the concentration of NSE at 6 h, 24 h, and 72 h postoperatively were significantly lower in the RIPC group. However, neither the MMSE nor the MoCA revealed significant between-group differences in postoperative cognitive performance at 7 days, 3 months, and 6 months after surgery.

**Conclusion:**

In patients undergoing CPB-assisted cardiac surgery, RIPC attenuated brain damage as indicated with the decreased release of brain damage biomarker S100-β and NSE.

**Clinical trial registration:**

[ClinicalTrials.gov], identifier [NCT01231789].

## Introduction

Neurological and neurobehavioral disorders are common complications after cardiopulmonary bypass (CPB)-assisted cardiac surgeries, including delirium, postoperative cognitive decline (POCD), and dementia. Postoperative cognitive dysfunction (POCD) is a common clinical complication with impacts on a wide array of cognitive domains, including attention, memory, executive function, and information processing speed (1). According to a recent meta-analysis, the prevalence of POCD following cardiac surgery was 28% between the first and fourth months postoperatively and 22% between the sixth and twelve months postoperatively (2), which is associated with disability and a poor postoperative quality of life (3–5). In addition, early cognitive decline following cardiac surgery may persist or become permanent, posing a significant risk of morbidity and mortality (6–8). Nonetheless, how to mitigate early-phase postoperative cognitive decline after cardiac surgery remains a major clinical challenge.

Remote ischemic preconditioning (RIPC) is a low-cost, non-invasive, and easy-to-use technique for attenuating ischemic organ injury by inducing transient sublethal episodes of ischemia and reperfusion in non-vital tissues (e.g., skeletal muscles), thereby enabling remote vital organs to tolerate a subsequent prolonged ischemic event (9–11). Some proof-of-concept trials demonstrated the cardiac and renal protective effects of RIPC in patients undergoing cardiovascular surgeries (12–15). RIPC is clearly a systemic phenomenon (11), which involves the activation of humoral and neuronal signaling pathways (16) and induces the release of circulating protective cells (17) and protective soluble factors (18–23). RIPC was reported to effectively delay the onset of cognitive decline in individuals with neurological comorbidities such as subcortical ischemic vascular dementia, cerebral small-vessel disease, and ischemic stroke (24–26). However, the efficacy of RIPC on neuroprotection in patients undergoing cardiovascular surgery remains controversial. Two studies failed to verify the neuroprotective effects of remote ischemic preconditioning in attenuating postoperative cognitive impairment in patients undergoing heart surgeries (27, 28), whether on CPB or not. In contrast, Hudetz et al. reported in a pilot study that RIPC prevented short-term deterioration of cognitive function after cardiac surgery (29).

Peripheral blood biomarkers convey information on a variety of pathological states (30). An elevated serum S-100 calcium-binding protein β (S100-β) level was associated with the severity of the brain injury (31), while the elevation of Neuron-specific enolase (NSE) indicated acute ischemic brain injury (32). Serum NSE and S100-β levels were also reported to be elevated in patients with neurological complications following cardiac surgeries (33). The 2 most commonly studied blood-based biomarkers of brain injury after cardiac arrest are S100-β and neuron-specific enolase (NSE) (34–36).

Therefore, the objective of the current study was to test the hypothesis that RIPC, as compared to standard care, attenuates brain injury as measured by the dynamic changes of S100-β and NSE in patients undergoing CPB-assisted CABG or valve surgery. In addition, we sought to determine if RIPC ameliorated postoperative neurocognitive dysfunction as assessed with Mini-mental State Examination (MMSE) and the Montreal Cognitive Assessment (MoCA).

## Materials and methods

### Trial design

A multi-center, parallel-group, superior, randomized controlled clinical trial was conducted in 3 cardiac surgery centers in China. The Department of Anesthesiology and Perioperative Medicine at Xijing Hospital was the trial coordinating center, which was responsible for trial design, generating randomization sequence, data verification, maintenance, and analysis. The trial protocol was approved by the Ethics Committee or Institutional Review Board of the coordinating center and the participating centers (Tianjin Chest Hospital and Henan provincial People’s Hospital). The investigation was conducted according to the Declaration of Helsinki and relevant Chinese laws. After approval by Institutional Review Board, the trial was registered on ClinicalTrials.gov (Identifier: NCT01231789). The trial was conducted in accordance with the principles of Good Clinical Practice. Data were monitored and audited by independent investigators from the coordinating center. Reporting follows the guidance of CONSORT recommendations. The full protocol with the statistical plan was attached in Supplementary material. All of the authors verify the accuracy and completeness of the data and the analysis.

### Participants

Patients were included when they were 18–75 years old and scheduled for CPB-assisted CABG or valve surgery. We excluded patients with prior cardiac surgery history or those who underwent urgent or emergent surgery. Patients with hepatic dysfunction (Child-Pugh Score: Class C), pulmonary disease (forced expiratory volume in 1 s < 40% the predicted value), renal failure (estimated glomerular filtration rate < 30 ml/min/1.73 m^2^), or cardiac dysfunction [ejection fraction (EF) < 40%] were excluded. Patients with severe comorbidities that interfere with outcome measurement or RIPC implementation (i.e., an episode of stroke within 3 months or peripheral vascular disease affecting the upper limb) were excluded. In addition, patients with any risk factors that could influence the cognitive function evaluation were excluded. These included a lack of formal education (be educated for less than 7 years); visual or auditory impairment; preoperative cognitive dysfunction (MMSE score < 24 or MoCA score < 26); or a history of mental illness. Eligible patients were enrolled in the trial after written informed consent was provided.

### Randomization, intervention, and blinding

Patients were randomized with a 1:1 ratio into RIPC or Control group. Randomization was performed centrally at the coordinating center and was stratified according to participating center. The generated randomization sequence was sealed in envelopes and sent to each site. To conceal allocation, investigators were allowed to open the envelope right before implementing of RIPC after the enrolled patient entered the pre-anesthesia preparation room. After catheterization in the left radial artery under local anesthesia for baseline blood pressure measurement, RIPC was induced by three cycles of right upper limb ischemia and reperfusion (To ensure the effect of remote ischemic preconditioning, the cuff should be inflated to high enough pressure to induce limb ischemia. Therefore, for patients with baseline systolic pressure lower than 150 mmHg, it was inflated to 200 mmHg; while for patients with comorbidities of hypertension (baseline systolic pressure higher than 150 mmHg), the cuff was inflated to 50 mmHg higher than the baseline systolic blood pressure for 5 min) by an appointed investigator in each center who was aware of the study-group allocation. For patients allocated to the control group, the blood pressure measurement cuff was inflated to a baseline diastolic pressure level for 5 min to generate a non-ischemic upper-limb compression, thus blinding the patient to the greatest extent possible. During the entire RIPC procedure, the surgical drapes were covered to blind the clinicians. Therefore, the anesthesiologist, surgeons, intensive care unit physicians, nurses, central lab personnel, and other study investigators were blinded to the treatment allocations.

### Procedures

Anesthesia management, surgical procedures and perioperative management followed the institutional routine at each site. Briefly, arterial blood pressure, central venous pressure, electrocardiographic tracings, and nasopharyngeal temperature were monitored continuously. Anesthesia was induced with bolus injection of midazolam, etomidate and sufentanil and maintained with sevoflurane, sufentanil and rocuronium. Rocuronium was used to facilitate endotracheal intubation. During surgery, the bispectral index was maintained at 40–60. Surgery was performed using standard non-pulsatile CPB under mild hypothermia (core temperature range at 28–30°C). The blood cardioplegia was adopted for myocardial protection. During cardiopulmonary bypass (CPB), mean arterial pressure was maintained between 50 and 70 mmHg, and mixed venous oxygen saturation was > 65%. After the procedure and wean off the CPB, the protamine was administrated to neutralize the effect of heparin. Blood samples were collected before anesthesia induction (Time Point 1, TP1), before cardiopulmonary bypass (TP2), at the end of the surgery (TP3) and 6 h (TP4), 24 h (TP5), 48 h (TP6), 72 h (TP7) after surgery. Blood samples were inverted gently several times, allowed to clot for 30 min at room temperature, and centrifuged at 1,500 rpm for 15 min to obtain serum. Serum samples were then stored at −80°C before analysis. The S100-β and NSE were quantified in batches at the clinical central laboratory of Xijing Hospital using an electrochemiluminescence-based one-step enzyme immunoassay (Elecsys 2010; Roche Diagnostics, United Kingdom).

### Outcomes

The primary endpoint was S100-β concentrations at the 6-h post-surgery. Secondary endpoints included S100-β levels at the remaining time points, the NSE levels, and postoperative neurocognitive function scores assessed with the Chinese version Mini-mental State Examination (MMSE) and Montreal Cognitive Assessment (MoCA) (37, 38). The MMSE test includes simple questions and problems in several areas: the time and place of the test, repeating lists of words, arithmetic such as the serial sevens, language use and comprehension, and basic motor skills. The MoCA test was characterized by good concurrent validity and could detect cognitive impairment in different neurological disorders, which was used to assess different types of cognitive abilities, including orientation, short-term memory or delayed recall, executive function or visuospatial ability, language abilities, abstraction, animal naming, attention and clock-drawing test. The baseline cognitive function was evaluated 1 day before the surgery. The postoperative cognitive function assessment was conducted at 7 days, 3 months, and 6 months post-surgery. When postoperative MMSE and MoCA scores were reduced more than 1 standard deviation (SD) as compared to the baseline value, POCD was diagnosed. The clinical indicators of postoperative quality of recovery (length of intensive cardiac care unit stay, emergence time, extubation time, and ventilation time) were compared between RIPC and the control group and considered an exploratory analysis.

### Statistical analysis

According to our preliminary experiments (30 cases), the serum S100-β level at 6 h post-surgery was 48.12 (mean) pg/ml in the RIPC group and 66.42 (mean) pg/ml in the control group, with a pooled standard deviation of 32.5. The sample size was calculated using PASS (Product Application and Support Software, PASS 15.0). At a power of 0.8 and a two-sided significance level of 0.05, at least 51 patients were required in each intervention group. Finally, 120 patients were enrolled for randomization to account for possible dropouts. The continuous variables were expressed as mean and standard deviation (SD) or median (interquartile range) as appropriate. The discrete variables were presented as frequencies and percentages. Baseline characteristics and the clinical outcome of the patients in both RIPC and control groups were compared *via* the independent *t* test, chi-square test or Fisher exact tests as appropriate. Analysis of covariance was adopted for the primary outcome analysis with the treatment group and randomized stratification parameters were used as factors, with baseline values serving as covariates. The primary analysis was based on a modified intention-to-treat (mITT) principle. No imputation was performed for missing data.

For the secondary outcomes, considering that the measures were taken repetitively, the effects of the intervention on S100-β, NSE, MMSE and MoCA, respectively, were analyzed with linear mixed effect models. The response variables were S100-β, NSE, MMSE and MoCA, respectively, with the grouping information, measurement times and their interactions as explanatory factors. The log-transformation was applied to S100-β, NSE, MMSE and MoCA to adjust for the skewness in variable distributions. The impact of different interventions on the primary endpoint (S100-β level at the 6 h post-surgery) was revealed on the adjustment of random variations for different study subjects and time points. Similar logic was followed in interpretation of secondary responses including S100-β at all times measured, NSE, MMSE and MoCA. Adjusting for temporal trends and subject random intercepts, the grouping is shown to affect both the S100-β and NSE levels. The measurements for MMSE and MoCA are majorly different among individuals and fluctuate with times considered. The inclusion of fixed effects and random effects is determined upon comparison of nested models. This is in line with the step-wise variable selection procedure with the most optimal fit indicated by the Akaike Information Criterion (AIC). Missing data for secondary endpoints were imputed using the Last Observation Carried Forward method.

*Post hoc* analyses for the primary endpoint were performed in subgroups according to the types of cardiac surgery and age. No adjustments were conducted for the multiple comparisons in the *post hoc* analyses. Thus, they were considered exploratory. No imputation was performed for missing data in *post hoc* analyses. Two-sided hypothesis tests at a 5% significance level are carried out throughout this study. All statistical analyses were performed using R software (version 4.1.2, R Foundation for Statistical Computing, Vienna, Austria).

## Results

### Study population

**FIGURE 1:**
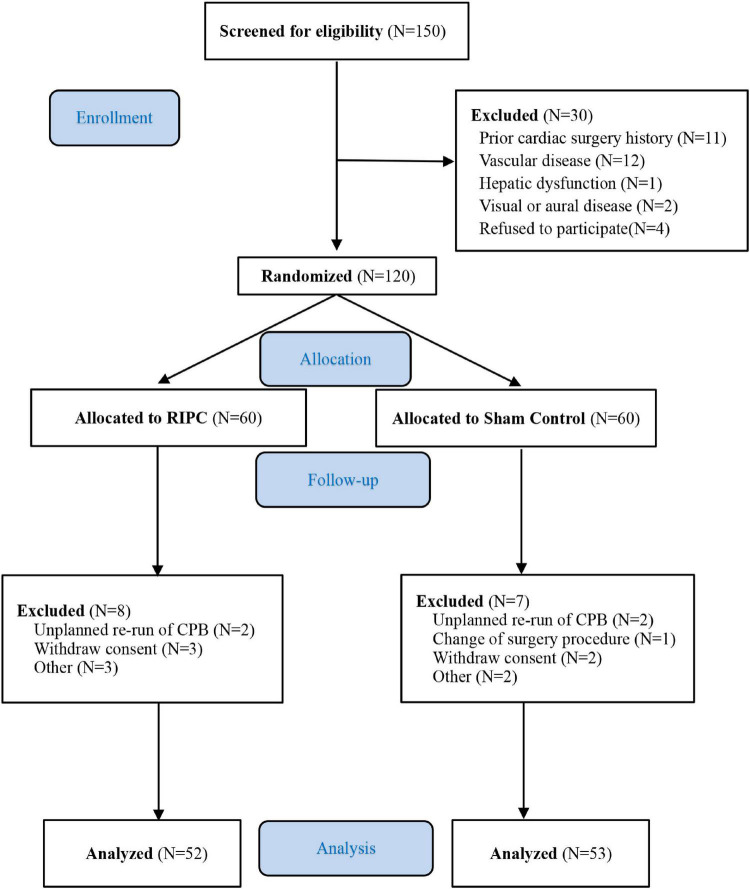
CONSORT flow diagram.

From June 2009 to August 2013, 150 patients were screened. Among them, 30 patients were ineligible due to factors such as a prior cardiac surgery history (*n* = 11), peripheral vascular disease (*n* = 12), hepatic dysfunction (*n* = 1), visual or auditory disorder (*n* = 2), or refusal refused to participate (*n* = 4). Final randomization of 120 patients scheduled for elective on-pump CABG or valvular surgery. Fifteen patients were excluded from the final data analysis based on the modified ITT, due to unplanned repetition of CPB, change of surgery procedure, the withdrawal of informed consent, and contaminated blood samples. Ultimately, 53 patients in the control group and 52 in the RIPC group completed the follow-up evaluation (Figure 1). The clinical and baseline characteristics of the 105 patients were presented in Table 1. There were no statistically significant differences in all baseline characteristics. The mean age was 48.6 years (SD 11.3) in the RIPC group and 49.0 years (SD 12.7) in the control group. Female patients accounted for 37.3% in the RIPC and 32.1% in the control group. The rates of comorbidities, medication history, operation type, CPB duration, and aortic cross-clamping time were comparable between the two groups.

**TABLE 1:** Baseline characteristics of the patients and surgical characteristics.

Characteristics	RIPC *N* = 52	Control *N* = 53
Patients		
Age — yr	48.6 ± 11.3	49.0 ± 12.7
Male sex — no./total no. (%)	33/52 (62.3)	36/53 (67.9)
Weight — kg	66.3 ± 12.0	65.9 ± 10.7
Height — cm	167.5 ± 6.3	168.3 ± 6.4
Prior diagnoses — no./total no. (%)		
Unstable angina	6/52 (11.5)	14/53 (26.4)
Diabetes mellitus	5/52 (9.6)	6/53 (11.3)
Hypertension	10/52 (19.2)	12/53 (22.6)
Drug history— no./total no. (%)		
Dopamine	45/52 (86.5)	38/53 (71.7)
Adrenalin	26/52 (50.0)	23/53 (43.4)
Lidocaine	9/52 (17.3)	7/53 (13.2)
Surgery		
Valve Replacement— no./total no. (%)	37/52 (71.2)	40/53 (75.5)
CABG— no./total no. (%)	15/52 (28.8)	13/53 (24.5)
Coronary artery lesions— no./total no. (%)		
1	4/52 (7.7)	3/53 (5.7)
2	4/52 (7.7)	1/53 (1.9)
3	4/52 (7.7)	6/53 (11.3)
4	2/52 (3.8)	3/53 (5.7)
5	1/52 (1.9)	0/53 (0)
Vavle lesions— no./total no. (%)		
1	23/52 (44.2)	28/53 (52.8)
2	13/52 (25.0)	7/53 (13.2)
3	2/52 (3.8)	6/53 (11.3)
CPB time — min	114.8 ± 43.2	118.4 ± 45.5
Myocardium ischemia time — min	64.2 ± 28.5	65.4 ± 28.8

Data are presented as n or mean (SD).

CPB, cardiopulmonary bypass; CABG, coronary artery bypass grafting.

### Primary outcomes

Elevated serum level of brain-derived proteins S-100 calcium-binding protein B (S100-β) was used as an early marker of cerebral damage. In this study, the preoperative baseline S100-β levels were within the normal range: 13.95 [95% confidence interval (CI), 11.64–16.26] pg/ml in the RIPC group and 12.40 (95% CI, 10.09–14.71) pg/ml in the control group (*p* = 0.333). At 6 h after surgery, S100-β was significantly lower in the RIPC group than that in the control group [43.17 (95% CI, 37.07–49.27) pg/ml in the RIPC group *vs* 77.92 (95% CI, 46.85–108.99) pg/ml in the control group, *p* = 0.035]. After adjusting for S100-β baseline levels, the least squares means of the two groups remained statistically different[50.75 (95% CI, 37.08–64.40) pg/ml in the RIPC group and 70.48 (95% CI, 56.84–84.10) pg/ml in the control group, *p* = 0.036] (Table 2).

**TABLE 2:** Differences in S100-β at 6 h post-surgery between remote ischemic preconditioning and control groups.

Time	RIPC	Control (Sham RIPC)	*P-value*
Pre-surgery†	13.95 (11.64–16.26)	12.40 (10.09–14.71)	0.333
6 h after surgery †	43.17 (37.07–49.27)	77.92 (46.85–108.99)	0.035
6 h after surgery ‡	50.75 (37.08–64.40)	70.48 (56.84–84.10)	0.036
6 h after surgery ¶	45.21 (33.93–56.50)	61.40 (50.24–72.60)	0.006
6 h after surgery $	65.94 (24.94–106.90)	96.68 (54.93–138.40)	0.333
6 h after surgery &	50.50 (40.33–60.70)	63.01 (53.04–73.00)	0.027
6 h after surgery #	43.52 (9.41–77.60)	79.96 (41.31–118.6)	0.984

†Data are mean (95% Confidence Interval) S100-β (pg/ml).

‡Data are least-squares means (95% Confidence Interval) S100-β (pg/ml).

¶Data are least-squares means (95% Confidence Interval) S100-β (pg/ml) in valvular surgery.

$Data are least-squares means (95% Confidence Interval) S100-β (pg/ml) in CABG.

&Data are least-squares means (95% Confidence Interval) S100-β (pg/ml) in the group under the age of 60.

#Data are least-squares means (95% Confidence Interval) S100-β (pg/ml) in the age group of 60 and above.

No imputation was performed for missing data.

### Secondary outcomes

**FIGURE 2:**
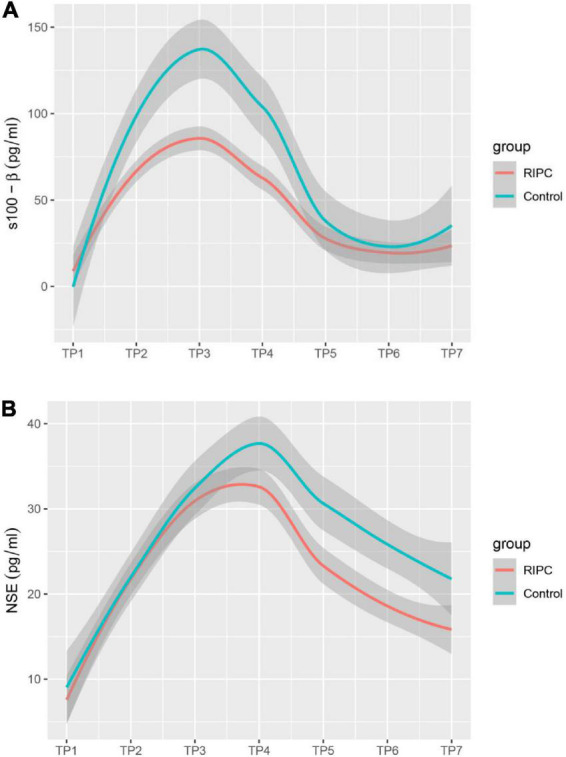
Estimated change trend in brain injury markers between RIPC group and control group across time points. S100-β **(A)**; S-100 calcium-binding protein B. NSE **(B)**; neuron specific enolase. Shaded areas indicate 95% confidence interval.

**FIGURE 3:**
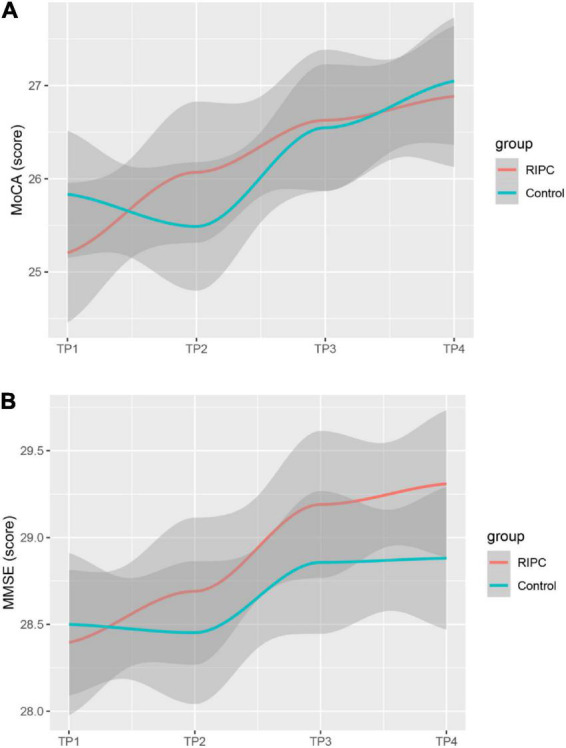
Estimated change trend in postoperative cognitive function between RIPC group and Control group across time points. Shaded areas indicate 95% confidence interval. MoCA **(A)**; Montreal Cognitive Assessment. MMSE **(B)**; Mini-mental State Examination.

**FIGURE 4:**
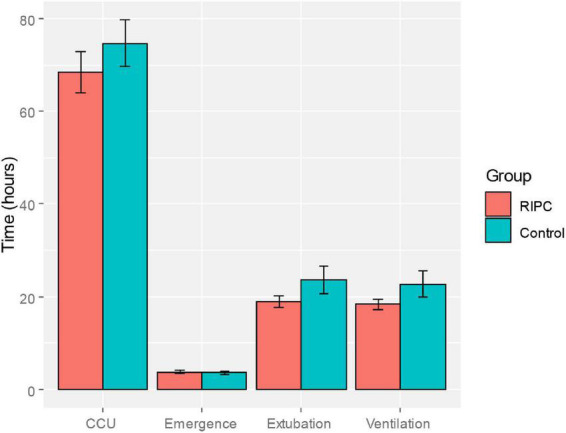
Comparison of clinical indicators of postoperative recovery (length of intensive cardiac care unit stay, emergence time, extubation time and ventilation time) between the RIPC group and the control group.

In both groups, there was a transient increase in S100-β at the end of surgery (TP3). However, the serum level of S100-β in the RIPC group was significantly lower at the end of the surgery, 6 h and 24 h compared to that of the control group (*P* < 0.05) (Table 3 and Figure 2A). After adjusting for the variation in different time points and individual participants, the concentration of S100-β in the control group remained greater than that of the RIPC group. Similarly, a temporary raise in NSE was detected in both groups shortly following surgery. Subsequently, the NSE in the RIPC group was significantly lower from 6 h to 72 h following surgery (Table 3 and Figure 2B). After adjusting for the variation of time points and individual participants, the level of NSE in the RIPC remained significantly lower than that of the control group. There was an interaction between intervention and time in the change of NSE, whereas the interaction effects were not observed for the change of S100-β concentration (Table 4). Patients were followed up for 6 months, and their MoCA and MMSE scores were obtained at 4-time points. MoCA and MMSE exhibited significant temporal patterns (Table 4), but there is no indication of between-group differences (Table 5 and Figure 3). MMSE and MoCA scores at baseline had standard deviations of 1.545 and 2.605, respectively. Figure 3 depicted cognitive function differences between RIPC and control groups. Four patients in the control group and five in the RIPC group experienced postoperative cognitive dysfunction (POCD) within 7 days following surgery. Fortunately, all nine patients recovered cognitive function at 3 or 6 months. Figure 4 depicted other clinical outcomes (cardiac intensive care unit length of stay, time to emergence and extubation, and duration of mechanic ventilation) in each treatment arm. No significant differences were found between these two groups. Additional *post hoc* analysis revealed significantly reduced S100-β concentration at 6 h postoperatively in the RIPC group compared to that in the control group in the valvular surgery subgroup, but not in the CABG subgroup (Table 2).

**TABLE 3:** S100-β and NSE before and after remote ischemic preconditioning.

	RIPC group *N* = 52	Control group *N* = 53	Between-group *P-value*
S100-β (pg/ml)			
Overall differences	28.4 (16.7, 51.3)	31.8 (17.7, 60.8)	0.075
Pre-anesthesia induction (TP 1)	12.1 (8.88, 19.5)	10.1 (8.09, 16.2)	0.1400
Pre-cardiopulmonary bypass (TP2)	48.4 (28.9, 64.2)	38.4 (22.0, 61.7)	0.446
Surgery ending (TP3)	96.1 (75.3, 118)	137 (86.5, 231)	0.008
6 h after surgery (TP4)	42.2 (26.6, 53.2)	52.1 (31.8, 76.5)	0.041
24 h after surgery (TP5)	21.2 (17.2, 25.8)	28.0 (22.1, 35.9)	< 0.001
48 h after surgery (TP6)	21.8 (16.2, 28.1)	27.2 (19.0, 38.7)	0.065
72 h after surgery (TP7)	18.9 (13.4, 25.4)	24.5 (14.2, 31.5)	0.012
NSE (pg/ml)			
Overall differences	17.5 (11.7, 28.3)	22.2 (11.1, 34.6)	0.006
Pre-anesthesia induction (TP 1)	10.5 (7.53, 12.3)	9.34 (6.67, 12.8)	0.530
Pre-cardiopulmonary bypass (TP2)	10.9 (8.79, 14.4)	8.83 (6.86, 11.9)	0.021
Surgery ending (TP3)	33.7 (27.7, 48.2)	36.5 (28.9, 43.7)	0.780
6 h after surgery (TP4)	29.8 (25.0, 33.5)	35.2 (26.3, 40.2)	0.014
24 h after surgery (TP5)	21.2 (17.2, 25.8)	28.0 (22.1, 35.9)	< 0.001
48 h after surgery (TP6)	16.8 (13.4, 19.3)	25.4 (16.7, 31.1)	< 0.001
72 h after surgery (TP7)	15.4 (11.6, 17.9)	20.6 (16.7, 26.1)	< 0.001

Values indicate the median and interquartile range. TP, Time point. The S100-β and NSE were collected before induction of anesthesia (Time Point 1, TP1), before cardiopulmonary bypass (TP2), end moment of the surgery (TP3) and 6 h (TP4), 24 h (TP5), 48 h (TP6), 72 h (TP7) after surgery. The postoperative cognitive function was measured preoperatively (Time Point 1, TP1), and 7 days (TP2), 3 months (TP3), 6 months (TP4) after surgery.

**TABLE 4:** Fixed effect of linear mixed effect model for repeated measurement of outcomes.

	Linear model	Linear mixed effect model
S 100-β		
Estimated effect	0.225 (0.081)**	0.230 (0.100)*
Akaike Inf. Crit.	2212.723	1757.298
NSE		
Time*group (RIPC)	0.077 (0.014)***	0.063 (0.096)
Time*group (Control)	0.109 (0.014)***	0.124 (0.096)
Akaike Inf. Crit.	1524.232	784.2942
MoCA		
Estimated effect (time)	0.516 (0.115)***	0.516 (0.082)***
Akaike Inf. Crit.	1556.473	1467.063
MMSE		
Estimated effect (time)	0.009 (0.002)***	8.374e^–3^ (1.302e^–3^)***
Akaike Inf. Crit.	−1,058.705	−1,255.923

Values indicate the estimated effect and corresponding standard error (SE).

*p < 0.05.

**p < 0.01.

***p < 0.001.

**TABLE 5:** MoCA and MMSE scores before and after remote ischemic preconditioning.

	RIPC group	Control group	Between-group *P-value*
MoCA (scores)			
Group difference	27.0 (25.0, 28.0)	27.0 (25.0, 28.0)	0.867
Baseline (TP 1)	26.0 (24.0, 27.0)	26.5 (24.2, 27.0)	0.182
7 days after surgery (TP2)	27.0 (25.0, 28.0)	26.0 (24.0, 27.0)	0.208
3 months after surgery (TP3)	27.0 (26.0, 28.0)	26.0 (26.0, 27.8)	0.381
6 months after surgery (TP4)	27.0 (26.0, 28.0)	27.0 (26.0, 28.0)	0.865
MMSE (scores)			
Group difference	29.0 (28.0, 30.0)	29.0 (28.0, 30.0)	0.051
Baseline (TP 1)	29.0 (28.0, 29.0)	29.0 (28.0, 30.0)	0.609
7 days after surgery (TP2)	29.0 (28.0, 30.0)	29.0 (27.2, 29.8)	0.377
3 months after surgery (TP3)	29.5 (29.0, 30.0)	29.0 (28.0, 30.0)	0.079
6 months after surgery (TP4)	30.0 (29.0, 30.0)	29.0 (29.0,30.0)	0.031

Values indicate the median and interquartile range. TP, Time point. The MoCA and MMSE were collected preoperatively (Time Point 1, TP1), 7 days (TP 2), 3 months (TP3) and 6 months (TP4) after surgery.

## Discussion

In high-risk patients undergoing on-pump CABG and valve surgery, remote ischemic preconditioning applied prior to cardiac surgery attenuated neurological damage following surgery compared to the sham preconditioning, as indicated by the serum biomarkers. However, remote ischemic preconditioning did not affect the postoperative cognitive function and other clinical outcomes as compared with sham preconditioning.

RIPC exhibits neuroprotective effects in animal models of cerebral ischemia, it was reported that RIPC enhanced the recovery of cortical neuronal activity, preserved cerebral oxygen pressure and reduced cortical damage following hypothermic circulatory arrest (39–41). Nonetheless, two previous clinical trials exploring the neuroprotective effects of RIPC in patients undergoing cardiac surgery revealed disappointing results (27, 28). Using established test batteries, Meybohm et al. (28) tested cognitive function in patients undergoing cardiac surgery. The diagnosis of postoperative cognitive dysfunction was based on the standard deviation or composite z score in two or more cognitive domains in order to evaluate the changes in cognitive function of patients before and after surgery. This study failed to demonstrate that remote ischemic preconditioning had a protective effect on cognitive function after cardiac surgery. However, a summarized z-score analysis revealed a trend toward a greater decline in cognitive function in the sham control group. In addition, in this study (28), the demographics were significantly different in the remote ischemic preconditioning group and the control group. The participants allocated in the RIPC group were more likely to be elderly, female, had diabetes or hypertension, or received beta-blockers. Unbalanced baseline data may be one of the possible explanations for these unfavorable results. By using 6 neuropsychometric tests, including (language learning, forward and reverse digit span, digit sign substitution, trajectory making parts A and B), Joung et al. (27) assessed the protective effect of remote ischemic preconditioning on postoperative cognitive function in patients undergoing off-pump CABG. Cognitive impairment was defined as a decrease of more than 20% in scores on two or more tests 1 week after surgery relative to preoperative scores. According to these criteria, there was no significant difference between the RIPC group and the control group in the incidence of postoperative cognitive impairment. Why did RIPC fail to provide neuroprotective effects for patients undergoing cardiac surgery in these two clinical trials? It is possible that regular perioperative management is facilitated by availability guidelines and has been refined in cardiac surgery, yet certain protective measures, such as cardiopulmonary bypass, hypothermia and bispectral index monitoring are clinical routine. Therefore, additional protection exerted from RIPC is difficult to detect. In addition, co-morbidities and co-medication as confounders for cardioprotection may interfere with the effect of RIPC, aortic cross-clamp time duration to make the efficacy of protection more evident and propofol to interfere with RIPC in patients undergoing cardiovascular surgery. However, the capacity of clinical investigations to identify a confounding impact of a single co-morbidity or co-medication is poor, and the amount of clinical data available for ischemic and pharmacological conditioning is limited (42).

The secondary endpoints of our study were consistent with the above-mentioned two trials, the incidence of POCD and long-term cognitive function were not significantly different between the RIPC and the control group. However, early postoperative moderate neurological damage was difficult to detect and was associated with poor long-term outcomes (6–8). As a result, we chose biomarkers of brain injury as the surrogate primary outcome measure. Serum and cerebral-spinal fluid levels of neuron-specific or astrocyte-specific proteins can be used to gauge the severity of central nervous system injury. S100-β and NSE are two of the most powerful diagnostic markers for brain injury among the various injury biomarkers. S100-β and NSE levels were elevated in the early phase of neuronal damage and were linked with subsequent cognitive impairment (43). Consistent with our findings, RIPC significantly limited the release of serum S100-β and NSE at 6 h and 1 day after elective cervical decompression surgery (44). However, there was no difference between RIPC and control groups in terms of S100-β and NSE levels in patients with aneurysmal subarachnoid hemorrhage or severe Carotid artery stenosis following surgery (45, 46). We then speculated that the neuroprotective effect of RIPC is surgery-type dependent. The severity of pre-existing brain injury, such as more invasive cerebrovascular surgery, may disguise the neuroprotective effect of RIPC. Additional *post hoc* analysis found that RIPC was ineffective at reducing S100-β levels 6 h after surgery in the CABG subgroup (*p* = 0.333). Similar to our findings, two large-scale trials have shown no benefits of applying RIPC before CABG surgery (47, 48).

The translation of remote ischemic conditioning to patients remains challenging (49). Experimental studies on the long-term effects of adjunctive cardioprotection beyond reducing infarct size, namely on repair, remodeling, and mortality are lacking. Adequate phase II dosing and timing studies are required when rushing from promising proof-of-concept trials to larger clinical outcome trials. Severe flaws in the design and conduct of clinical trials have also largely contributed to the failure to translate cardioprotection into clinical practice (50, 51). In addition, the clinical translation of RIPC to improve cognitive function after cardiac surgery also faces difficulties. In the present study, there was no significant difference in long-term postoperative cognitive function and other clinical outcomes between the RIPC and control groups. Contrary to expectations, although lowering the release of neurological damage biomarkers, RIPC appeared to have little effect on the clinical outcomes after cardiac surgery. Similarly, two adequately powered clinical trials failed to demonstrate any beneficial effect of RIPC on clinical outcomes in pediatric (52) and adult (53) patients. In our trial, we revealed that RIPC dramatically lowered the rise of S100-β and NSE, these two prognostic markers have been shown to correlate with the severity of cognitive dysfunction after cardiac surgery. Consequently, it is reasonable to conclude that RIPC can ameliorate postoperative cognitive dysfunction to some extent; however, the effect of RIPC may not be strong enough to alter the long-term cognitive outcomes. Additionally, age is a leading risk factor associated with postoperative cognitive dysfunction after cardiac surgery, older patients are more susceptible to postoperative cognitive dysfunction than younger patients (54, 55). In the current study, the average age of the participants in the current study was 48 years, indicating a relatively lower-risk patient population. Patients were separated into two strata: non-geriatric patients (age < 60 years) and geriatric patients (age ≥ 60 years) in the *post hoc* subgroup analysis. The effect modification was assessed by including an interaction term between age and trial group in the analysis. It was demonstrated that RIPC lowered the level of S100 β at 6 h after surgery in both age subgroups. The decrease in S100 β was not statistically different between the age ≥ 60 years subgroup. However, spurious negative effect modification effects of age may arise regrading the relatively small sample size.

Our study has some limitations. Firstly, only 105 patients were included in the final analysis, which is a relatively small sample size despite having sufficient statistical power. In the *post hoc* analysis, RIPC was ineffective at reducing S100-β levels at 6 h after surgery in the CABG subgroup, which was likely due to the small sample size and insufficient power of this subgroup. Second, the incidence of delirium was not assessed in our study. The symptoms of delirium may occur prior and are closely related to POCD. Third, as the RIPC was performed prior to anesthesia, the patient could perceive the cuff’s inflation and pressure. We did our utmost to blind the patient, but we cannot rule out the possibility that he or she could figure out the difference in inflation pressure through communications with other enrolled patients. However, since the primary endpoint was the S100-β concentration, which was measured by the blinded clinical central lab personnel and was relatively objective, thus the concerns regarding placebo effects were considerably reduced. Fourth, the choice of serum biomarkers as the primary endpoint may affect the extrapolation of study results. As the beneficial effect of RIPC on surrogate outcomes may not immediately translate into the clinical outcomes that are important to patients (56, 57). Lastly, the included patients are a historical collective, beyond that, there are no follow-up data beyond the 6 months.

## Conclusion

In conclusion, in our multicenter randomized clinical trial, upper-limb RIPC reduced the postoperative elevation of brain injury biomarkers S100-β and NSE in patients undergoing elective CPB-assisted coronary artery bypass grafting or valve surgery. However, there was no significant difference in postoperative cognitive performance between RIPC and the control group at 7 days, 3 months, and 6 months after surgery.

## Data availability statement

The original contributions presented in this study are included in the article/Supplementary material, further inquiries can be directed to the corresponding author/s.

## Ethics statement

The studies involving human participants were reviewed and approved by the Ethics Committee of Xijing Hospital (No: XJIRB-20090625-2). The patients/participants provided their written informed consent to participate in this study.

## Author contributions

SZ contributed to the drafting the article. CL and HD contributed to the conception and design of the study. WL, PO, JH, and JZ contributed to the data acquisition. SZ and ZZ contributed to the analysis and interpretation of data. CL contributed to the reviewing final manuscript. All authors contributed to the article and approved the submitted version.
