# Supplementary material for: Neuroprotective effect of remote ischemic preconditioning in patients undergoing cardiac surgery: A randomized controlled trial
Source: Front Cardiovasc Med. 2022 Sep 6;9:952033. doi: 10.3389/fcvm.2022.952033 (PMC9485807; doi:10.3389/fcvm.2022.952033)
Supplement: Supplementary file 1 [file Presentation_1.pdf]

Study Protocol

Background

Neurological and neurobehavioral disorders are common complications of cardiopulmonary bypass (CPB) surgeries, such as on-pump coronary artery bypass grafting surgery and valve replacement surgery. They are likely to result in disability and a poor postoperative quality of life(1-3). Both disorders extend the intensive care unit (ICU) and hospital length of stay, leading to increased hospitalization costs(4). Such postoperative neurocognitive dysfunction could be attributed to multiple underlying perioperative factors (e.g., hypoperfusion, thromboembolism, and cerebral inflammation)(5). Lots of medical measures were attempted to reduce its incidence and made mighty advances, such as intraoperative hypothermia(6), a neuroprotective agent like NXY-059(7), and hemodilution(8, 9).

Transient sublethal episodes of ischemia in non-vital tissues (e.g., skeletal muscles) have been shown to enhance the tolerance of remote vital organs (e.g., the heart, brain and kidney) to the subsequent prolonged ischemia/reperfusion injuries in a number of clinical conditions; a phenomenon known as remote ischemic preconditioning (RIPC). RIPC is a strategy to protect the target organs by inducing a short period of ischemia-reperfusion in the distal tissues(10). Increasing evidence suggests that RIPC can reduce the incidence of complications such as acute kidney injury and myocardial injury after cardiac surgeries (11, 12). In addition, RIPC could delay the development of cognitive decline in patients with organic disorders, such as subcortical ischemic vascular dementia, cerebral small-vessel disease and ischemic stroke(13-15). However, the effects of RIPC on postoperative cognitive dysfunction after cardiac surgery remain inconsistent.

Peripheral blood neurobiochemical markers of brain injury convey information about various pathological states(19). Among these makers, the increased serum S-100 calcium-binding protein B (S100-β) levels expressed in astrocytes were associated with the severity of the brain injury(20). Neuron-specific enolase (NSE) is another neurovascular function biomarker that is elevated in acute ischemic brain injury(21). Increased blood levels of NSE and S100-β have also been found in patients who sustained clinical neurological complications(22) after cardiac surgeries. Therefore, this randomized controlled study aims to explore the influence of RIPC on early brain injury after cardiac surgeries via a comparison of the dynamic changes in these two markers. The Mini-mental

State Examination (MMSE) and the Montreal Cognitive Assessment (MoCA) also looked into postoperative neurocognitive dysfunction.

Methods

Study population

The study is designed as a multicenter, prospective, randomized, double-blind controlled trial (**Figure. 1**). Adult patients with coronary or valve disease undergoing elective cardiac surgery at Xijing Hospital, Tianjin Chest Hospital, and Henan provincial People's Hospital will be eligible for study enrollment. Following the process of primary triage, they will be approached by a study nurse who will provide detailed information about the trial. Written informed consent will be obtained from all patients prior to enrollment. All included patients signed written informed consent. The criteria were: Patients who suffered from cardiac disease and were about to undergo CABG or valve replacement with a CPB system; under the age of 75 years old and above 18; without diabetes mellitus, hepatic, renal, pulmonary or peripheral vascular disease. Exclusion criteria were: urgency surgery; cardiac surgery experience; education time below 7 years; ejection fraction(EF)< 40%; the visual or aural disease that will affect the cognitive tests; an episode of cerebral vascular disease within 3 months; twice CPB in the surgery. All participants were asked to give informed assent by themself, or they were next of kin. Patients were randomly assigned to either the RIPC or control group before cardiac surgery.

Ethics

This study was performed following the Declaration of Helsinki and relevant Chinese laws. The study received the Ethics Committee of Xijing Hospital and registered on ClinicalTrials.gov. The approval number was NCT01231789. This protocol complies with CONSORT guidelines(1).

Sample size calculation

According to our preliminary experiments (30 cases), the serum S100-β level at 6 hours post-surgery was 48.12±20.56 (mean±SD)pg/ml in the RIPC group and 66.42±39.88 (mean±SD) pg/ml in the control group, with a pooled standard deviation of 32.51 and at the 5% level of significance. The sample size was calculated using PASS (Product Application and Support Software, PASS 15.0). At a power of 0.8 and a two-sided significance level of 0.05, at least 51 patients were required in each intervention group. Finally, 120 patients were enrolled for randomization to

account for possible dropouts.

Randomization, Intervention and blinding

Patients were randomized with a 1:1 ratio into RIPC or Control group. Randomization was performed centrally at the coordinating centre and was stratified according to participating centre. The generated randomization sequence was sealed in envelopes and sent to each site. To conceal allocation, investigators were allowed to open the envelope right before implementing RIPC after the enrolled patient entered the pre-anaesthesia preparation room. After cannulating in the left radical artery under local anaesthesia for baseline blood pressure measurement, RIPC was induced by three cycles of right upper arm ischemia and reperfusion (To ensure the effect of remote ischemic preconditioning, the cuff should be inflated to a high enough pressure to induce limb ischemia. Therefore, for patients with baseline systolic pressure lower than 150 mmHg, it was inflated to 200 mmHg; while for patients with comorbidities of hypertension (baseline systolic pressure higher than 150 mmHg), the cuff was inflated to 50 mmHg higher than the baseline systolic blood pressure for 5 minutes) by an appointed investigator in each centre who was aware of the study-group allocation. For patients allocated to the control group, the blood pressure measurement cuff was inflated to a baseline diastolic pressure level for 5 minutes to generate a non-ischemic upper-arm compression, thus blinding the patient to the greatest extent possible. The surgical drapes were covered during the entire RIPC procedure to blind the clinicians. Therefore, the anesthesiologist, surgeons, intensive care unit physicians, nurses, central lab personnel, and other study investigators were blinded to the treatment allocations.

Procedures

Anaesthesia management, surgical procedures and perioperative management followed the institutional routine at each site. Briefly, Arterial blood pressure, central venous pressure, electrocardiographic tracings, and nasopharyngeal temperature were monitored continuously. Anaesthesia was induced with bolus injection of midazolam, etomidate and sufentanil and maintained with sevoflurane, sufentanil and rocuronium. Rocuronium was used to facilitate endotracheal intubation. During surgery, the bispectral index was maintained at 40-60. Standard non-pulsatile CPB was instituted, and surgery was performed with mild hypothermia (core temperature range at 28-30℃). Blood cardioplegia was adopted for myocardial protection. Blood flow during CPB was adjusted to achieve a mean arterial blood pressure of 50-70 mmHg and a mixed venous oxygen saturation > 65%. After the procedure and wean off the CPB, Protamine was administrated to neutralize the effect of heparin. Blood samples were collected before anaesthesia induction (Time Point 1, TP1), before cardiopulmonary bypass (TP2), at the end of the surgery (TP3) and 6h (TP4), 24h (TP5), 48h (TP6), 72h (TP7) after surgery. All of the blood

samples were centrifuged. Plasma was snap frozen and stored at -80℃. The S100-β and NSE were quantified in batches at the central clinical laboratory of Xijing Hospital using an electrochemiluminescence-based one-step enzyme immunoassay(Elecsys 2010; Roche Diagnostics, UK).

Outcomes

The primary endpoint was S100-β concentrations at the 6-hour post-surgery. Secondary endpoints included S100-β levels at the remaining time points, the NSE levels, and postoperative neurocognitive function scores assessed with the Chinese version Mini-mental State Examination (MMSE)(22) and Montreal Cognitive Assessment (MoCA)(23). The baseline cognitive function was evaluated one day before the surgery. The postoperative cognitive function assessment was conducted at 7 days, 3 months, and 6 months post-surgery. Postoperative cognitive dysfunction was defined as a 1 SD or more decline in MMSE and MoCA tests anytime after surgery compared with the baseline score.

Statistical analysis

The continuous variables were expressed as appropriate as mean and standard deviation (SD) or median (interquartile range). The discrete variables were presented as frequencies and percentages. Baseline characteristics and the clinical outcome of the patients in both RIPC and control groups were compared via the independent t-test, chi-square test or Fisher exact tests as appropriate. Analysis of covariance was adopted for the primary outcome analysis with the treatment group, and randomized stratification parameters were used as factors, with baseline values serving as covariates. The primary analysis was based on a modified intention-to-treat (mITT) principle. No imputation was performed for missing data. For the secondary outcomes, considering that the measures are taken repetitively, the effects of the intervention on S100-β, NSE, MMSE and MoCA respectively were analyzed with linear mixed effect models. The response variables were S100-β, NSE, MMSE and MoCA respectively, with the grouping information, measurement times and their interactions as explanatory factors. The log-transformation was applied to S100-β, NSE, MMSE and MoCA to adjust for the skewness in variable distributions. The impact of different intervention on the primary endpoint (S100-β level at the 6h post-surgery) was revealed on the adjustment of random variations for different study subjects and time points. Similar logic was followed in interpretation of secondary responses including S100-β at all times measured, NSE, MMSE and MoCA. Adjusting for temporal trends and subject random intercepts, the grouping is shown to affect both the S100-β and NSE levels. The measurements for MMSE and MoCA are majorly different among individuals and fluctuate with times considered. The inclusion of fixed effects and random effects is determined upon the comparison of nested models.

This is in line with the step-wise variable selection procedure with the most optimal fit indicated by the Akaike Information Criterion (AIC). Missing data for secondary endpoints were imputed using the Last Observation Carried Forward method. Post hoc analyses for the primary endpoint were performed in subgroups according to the types of cardiac surgery. No adjustments were conducted for the multiple comparisons in the post hoc analyses. Thus, they were considered exploratory. No imputation was performed for missing data in post hoc analyses. Two-sided hypothesis tests at a 5% significance level are carried out throughout this study. All statistic analyses were performed using R software (version 4.1.2, R Foundation for Statistical Computing, Vienna, Austria).

Discussion

Initially, ischemia preconditioning was presented in the form of local ischemia preconditioning. In 1986, C E Murry found that exposure of the circumflex coronary artery territory to brief periods of ischaemia before 40 min of complete ischaemia substantially reduced the extent of infarction after the restoration of blood flow(2). This is the first time local ischemia preconditioning was suggested to have a role in ischemia-reperfusion injury. However, because of the limitation of the application, local ischemia preconditioning was deemed too hard to use in clinical. The emergence of remote ischemia preconditioning is expected to solve this problem. Karen Przyklenk reported that brief episodes of ischemia in one vascular bed protect remote, virgin myocardium from subsequent sustained coronary artery occlusion in a canine model(3). Not only in myocardium protection, but remote ischemia preconditioning is also thought to work in neuroprotection. Kunjan R. Dave showed that remote ischemia preconditioning could provide neuroprotection in a rat model of asphyxial cardiac arrest(4). Nevertheless, no study reported that IRPC has the same function in humans.

In this study, we assumed that RIPC, induced by repeated transient upper limb ischemia and reperfusion, can reduce the serum level of S-100β and NSE in the patients undergoing CABG or valve replacement surgery. S-100β and NSE are predictive protein biomarkers reflecting the CNS injury, which can be measured in CSF and serum. S-100β protein is a Ca^{2+}-binding protein found in astroglial and Schwann cells and a few tumours. It was found elevated in all traumatic brain injuries (5) and non-traumatic brain injuries (Hydrocephalus, Aneurysmal, CNS infections)(6-9). NSE is a cytoplasmic glycolysis enzyme discovered in neurons and neuroendocrine cells(10, 11). The blood levels of NSE are associated with cerebral diseases with brain damage like ischemic stroke, meningoencephalitis and head injury(11, 12).

Postoperative cognitive dysfunction is the most frequent type of neurologic damage. It is considered a short-term decline in cognitive dysfunction lasting from a few days to a few weeks after surgery. However, in many cases, it becomes a permanent disorder that significantly impacts a patient's quality of life(13). Due to the increased overall cost, length of hospital stay, morbidity and mortality after cardiac surgery caused by postoperative dysfunction, many trials have been performed to reduce the incidence and clarify the mechanism. However, up to now, no valuable strategies have been established. Even more, there are many opposing views on preventing postoperative cognitive dysfunction. For example, Farag E and An J point out that more profound anaesthesia, either intravenous or Inhalation anaesthesia, can reduce the incidence of postoperative cognitive dysfunction(14, 15). However, in Lindholm ML's opinion, more profound anaesthesia aggravates the cognitive impairment(16). A similar situation happened in using general and regional anaesthesia, intravenous and inhalation anaesthesia and so on.

Though the pathophysiology and aetiology of POCD are not clear now, it is widely accepted that neuroinflammation may be the pathogenic basis of POCD. In Hudetz J. A's clinical study, the inflammation-relevant markers IL-6 and C-reactive protein were associated with the subsequent development of short- and medium-term impairment of cognitive functions after coronary artery surgery(17). As we know, the inhabitation of inflammatory reaction is considered to be why RIPC have the role of multiple organ protection. In Shimizu M's study, RIPC significantly reduced neutrophil, adhesion, phagocytosis and cytokine levels (18). So it may be considered that RIPC could decrease the incidence of POCD by restraining the nerve inflammation rate in the perioperative period.

The aetiology of brain injury following cardiac surgery is multifaceted, and not all of its potential triggers can be addressed in a single trial. Our study will focus on a strategy hypothesized to reduce the impact of ischemia on end-organ performance.

While the trial will enrol patients from cardiac surgical practice, the fact that the pathophysiology of brain ischemia is no different in higher-risk patients will allow for the results of this trial to be generalized to a broader surgical cohort. The preoperative implementation of the RIPC protocol may improve postoperative neurologic outcomes and meaningfully impact the standard of care in cardiac surgical patients.

Reference

1. Schulz KF, Altman DG, Moher D. CONSORT 2010 Statement: updated guidelines for reporting parallel group randomised trials. *Trials* (2010) 11:32. Epub 2010/03/26. doi: 10.1186/1745-6215-11-32. PubMed PMID: 20334632; PubMed Central PMCID: PMCPMC2857832.
2. Murry CE, Jennings RB, Reimer KA. Preconditioning with ischemia: a delay of lethal cell injury in ischemic myocardium. *Circulation* (1986) 74(5):1124-36. doi: 10.1161/01.cir.74.5.1124.
3. Przyklenk K, Bauer B, Ovize M, Kloner RA, Whittaker P. Regional ischemic 'preconditioning' protects remote virgin myocardium from subsequent sustained coronary occlusion. *Circulation* (1993) 87(3):893-9. doi: 10.1161/01.cir.87.3.893.
4. Dave KR, Saul I, Prado R, Busto R, Perez-Pinzon MA. Remote organ ischemic preconditioning protect brain from ischemic damage following asphyxial cardiac arrest. *Neuroscience letters* (2006) 404(1-2):170-5. doi: 10.1016/j.neulet.2006.05.037. PubMed PMID: 16781056.
5. Schafer BW, Heizmann CW. The S100 family of EF-hand calcium-binding proteins: functions and pathology. *Trends in biochemical sciences* (1996) 21(4):134-40. PubMed PMID: 8701470.
6. Beems T, Simons KS, Van Geel WJ, De Reus HP, Vos PE, Verbeek MM. Serum- and CSF-concentrations of brain specific proteins in hydrocephalus. *Acta neurochirurgica* (2003) 145(1):37-43. doi: 10.1007/s00701-002-1019-1. PubMed PMID: 12545260.
7. Herrmann M, Vos P, Wunderlich MT, de Bruijn CH, Lamers KJ. Release of glial tissue-specific proteins after acute stroke: A comparative analysis of serum concentrations of protein S-100B and glial fibrillary acidic protein. *Stroke; a journal of cerebral circulation* (2000) 31(11):2670-7. PubMed PMID: 11062293.
8. Lins H, Wallesch CW, Wunderlich MT. Sequential analyses of neurobiochemical markers of cerebral damage in cerebrospinal fluid and serum in CNS infections. *Acta neurologica Scandinavica* (2005) 112(5):303-8. doi: 10.1111/j.1600-0404.2005.00484.x. PubMed PMID: 16218912.
9. Wiesmann M, Missler U, Hagenstrom H, Gottmann D. S-100 protein plasma levels after aneurysmal subarachnoid haemorrhage. *Acta neurochirurgica* (1997) 139(12):1155-60. PubMed PMID: 9479422.
10. Marangos PJ, Schmechel DE. Neuron specific enolase, a clinically useful marker for neurons and neuroendocrine cells. *Annual review of neuroscience* (1987) 10:269-95. doi:

10.1146/annurev.ne.10.030187.001413. PubMed PMID: 3551759.
11. Snyder-Ramos SA, Bottiger BW. Molecular markers of brain damage--clinical and ethical implications with particular focus on cardiac arrest. *Restorative neurology and neuroscience* (2003) 21(3-4):123-39. PubMed PMID: 14530575.
12. Pfeifer R, Borner A, Krack A, Sigusch HH, Surber R, Figulla HR. Outcome after cardiac arrest: predictive values and limitations of the neuroproteins neuron-specific enolase and protein S-100 and the Glasgow Coma Scale. *Resuscitation* (2005) 65(1):49-55. doi: 10.1016/j.resuscitation.2004.10.011. PubMed PMID: 15797275.
13. Grape S, Ravussin P, Rossi A, Kern C, Steiner LA. Postoperative cognitive dysfunction. *Trends in Anaesthesia and Critical Care* (2012) 2(3):98-103. doi: 10.1016/j.tacc.2012.02.002.
14. An J, Fang Q, Huang C, Qian X, Fan T, Lin Y, et al. Deeper total intravenous anesthesia reduced the incidence of early postoperative cognitive dysfunction after microvascular decompression for facial spasm. *Journal of neurosurgical anesthesiology* (2011) 23(1):12-7. doi: 10.1097/ANA.0b013e3181f59db4. PubMed PMID: 21252704.
15. Farag E, Chelune GJ, Schubert A, Mascha EJ. Is depth of anesthesia, as assessed by the Bispectral Index, related to postoperative cognitive dysfunction and recovery? *Anesthesia and analgesia* (2006) 103(3):633-40. doi: 10.1213/01.ane.0000228870.48028.b5. PubMed PMID: 16931673.
16. Lindholm ML, Traff S, Granath F, Greenwald SD, Ekbom A, Lennmarken C, et al. Mortality within 2 years after surgery in relation to low intraoperative bispectral index values and preexisting malignant disease. *Anesthesia and analgesia* (2009) 108(2):508-12. doi: 10.1213/ane.0b013e31818f603c. PubMed PMID: 19151279.
17. Hudetz JA, Gandhi SD, Iqbal Z, Patterson KM, Pagel PS. Elevated postoperative inflammatory biomarkers are associated with short- and medium-term cognitive dysfunction after coronary artery surgery. *Journal of anesthesia* (2011) 25(1):1-9. doi: 10.1007/s00540-010-1042-y. PubMed PMID: 21061037.
18. Shimizu M, Saxena P, Konstantinov IE, Cherepanov V, Cheung MM, Wearden P, et al. Remote ischemic preconditioning decreases adhesion and selectively modifies functional responses of human neutrophils. *The Journal of surgical research* (2010) 158(1):155-61. doi: 10.1016/j.jss.2008.08.010. PubMed PMID: 19540519.

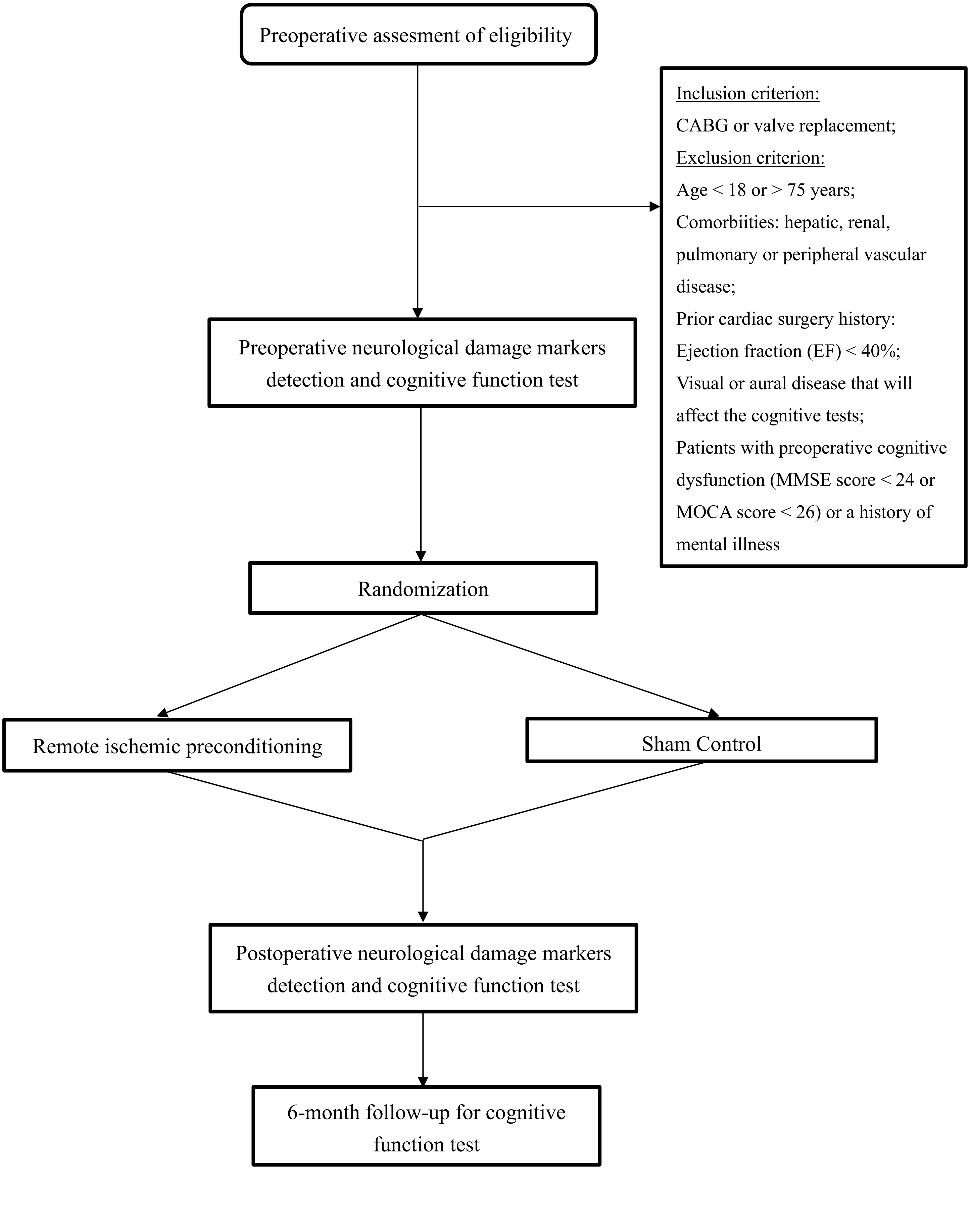
Preoperative assesment of eligibility
Inclusion criterion:
CABG or valve replacement;
Exclusion criterion:
Age < 18 or > 75 years;
Comorbiities: hepatic, renal, pulmonary or peripheral vascular disease;
Prior cardiac surgery history:
Ejection fraction (EF) < 40%;
Visual or aural disease that will affect the cognitive tests;
Patients with preoperative cognitive dysfunction (MMSE score < 24 or MOCA score < 26) or a history of mental illness
Preoperative neurological damage markers detection and cognitive function test
Randomization
Remote ischemic preconditioning
Sham Control
Postoperative neurological damage markers detection and cognitive function test
6-month follow-up for cognitive function test

Figure. 1 Flowchart depicting the screening, recruitment and randomization algorithm.
